# Circular RNAs as Diagnostic and Prognostic Indicators of Colorectal Cancer: A Pooled Analysis of Individual Studies

**DOI:** 10.3389/pore.2022.1610037

**Published:** 2022-03-17

**Authors:** Cong Long, Qiu-bo Xu, Li Ding, Li-juan Huang, Yong Ji

**Affiliations:** ^1^ Department of Clinical Laboratory, Jingjiang People’s Hospital, Taizhou, China; ^2^ Department of General Surgery, Jingjiang People’s Hospital, Taizhou, China

**Keywords:** colorectal cancer, circular RNA, diagnosis, prognosis, pooled analysis

## Abstract

**Background:** Circular RNAs (circRNAs) have proven as a special subset of endogenous RNAs that are implicated in the tumorigenesis of various cancers. This study sought to evaluate the role of circRNAs in the diagnosis and prognosis of colorectal cancer (CRC).

**Methods:** The online databases were searched for collecting relevant studies on circRNAs as diagnostic and prognostic biomarkers of CRC. Two researchers independently screened literature, extracted data, and evaluated the bias and risks of included studies. The diagnostic and prognostic indicators were merged and analyzed using STATA 12.0 software, and sources of heterogeneity were traced by the sensitivity analysis and the meta-regression test.

**Results:** A total of 29 articles representing 2639 CRC patients were included. The pooled sensitivity, specificity, and area under the curve (AUC) of circRNAs in differentiating CRC from non-tumor control were 0.75 (95% CI: 0.69–0.80) and 0.74 (95% CI: 0.69–0.78) and 0.81, respectively. The survival analysis showed that up-regulations of up-regulated circRNAs were significantly related to dismal survival in CRC patients (HR = 2.38, *p* < 0.001). A stratified analysis showed that the comprehensive diagnostic value of up-regualted circRNAs in CRC was higher than that of down-regualted circRNAs (AUC: 0.83 vs. 0.77; Z test, *p* < 0.05). The efficacy of tissue-derived circRNAs in the diagnosis of CRC was equal to that of plasma/serum-derived ones (AUC: 0.81 vs. 0.82; Z test, *p* > 0.05).

**Conclusion:** Abnormally expressed circRNAs as auxiliary biomarkers present underlying value in the diagnosis and prognosis prediction of CRC.

## Introduction

CRC as one of the most common malignant tumors of the digestive tract accounts for 10.2% of total cancer cases—18.1 million new cases worldwide in 2018—according to Global Cancer Statistics 2018 released by WHO, ranking the third (1). As with the 2015 China Cancer report, CRC ranks the fourth and third in morbidity and mortality in the nation, respectively (2). Due to the concealment of early symptoms of the disease, most of the patients initially visit a doctor until the middle and late stage (3). Along with the limited therapeutic effect and multiple relapses, the prognosis of the patients is very poor (4). Therefore, establishing an early diagnostic system for CRC, which means better prognosis and timely adoptions of new and effective therapies, is the preoccupation for reducing the mortality and ameliorating the prognosis. Though endoscopic biopsy combined with histopathology is the gold standard for its diagnosis, such an invasive examination cannot be easily accepted by some patients (5,6). The diagnostic efficacy of traditional biomarkers including carcinoembryonic antigen (CEA), carbohydrate antigen 19-9 (CA19-9) and CA72-4 are limited (4). A published meta-analysis has shown that the total sensitivity of CA19-9 in the diagnosis of CRC is only from 0.68 to 0.71 (a threshold of 5 μg/L to 10 μg/L), far from the requirements of clinical diagnosis and treatments (7). In this respect, searching for new markers is an important means of early diagnosis of CRC.

CircRNAs are a class of non-coding RNA featuring covalent bindings that form a closed loop with the 3′ and 5′ ends (8,9). CircRNAs, widely expressed in mammalian cells, are tissue-cell specific, structurally stable and sequence-conserved (10). Some types of circRNAs are proven to play roles in the transcription and expression of genes via multiple ways, which play an important role in cell cycle, cell aging and other physiological processes (10,11). Accumulating studies have shown that circRNAs are critical in the occurrence and development of malignant tumors (12,13). At present, many studies have reported that circRNAs have underlying clinical values in the early diagnosis and prognosis evaluation of CRC (14–42). In view of the fact that there are noticeable problems of single-item single-center trials, such as small sample size and large result bias, this study intends to systematically evaluate the potential application value of circRNAs in clinical diagnosis and prognosis evaluation of CRC through a PRISMA-compliant pair-wise meta-analysis.

## Materials and Methods

### Data Search Strategy

Retrieval data were extracted from papers published in English on PubMed, EMBASE, Web of Science and CNKI databases due November 1st, 2021. The key words encompassed “colorectal cancer”, “colorectal carcinoma”, “carcinoma of colon”, “colorectal neoplasms”, “circular RNA”, “circRNA”, “hsa circ”, “diagnoses”, “diagnosis”, “sensitivity”, “specificity”, “area under the curve”, “AUC”, “ROC curve”, “prognoses”, “prognosis”, “survival”, “overall survival”, “progression free survival”, “hazard ratio”, “OS”, “PFS” and “HR”. Besides, references attached to the paper were also searched manually to prevent omission of any eligible literature.

### Inclusion and Exclusion Criteria

Inclusion criteria were listed as follows: 1) case-control studies; 2) studies on the evaluation of diagnostic value and/or prognosis of circRNA in CRC; 3) data of true positive number (TP), false positive number (FP), false negative number (FN) and true negative number (TN) that could be obtained directly or indirectly to construct a 2 × 2 four-grid table for the diagnostic meta-analysis; 4) the prognostic observation indice was total survival time (overall survival, OS), or progression free survival (PFS), for outputting the hazard ratio (HR) and 95% confidence interval (CI) directly or indirectly. Exclusion criteria were defined as follows: 1) a study size of less than 20 cases; 2) data used for statistical analysis were insufficient or unavailable even after contacting original authors; 3) non-English language articles or low quality research.

### Data Extraction

All relevant literature was screened by two trained authors. The basic information was extracted independently: name of the first author, date of publication, study population, sample size, the control type, circRNA signatures, detection methods, reference genes, cut-off value settings, follow-up time, as well as values of sensitivity, specificity, AUC, HR, and 95% CI(s) and so forth.

### Quality Assessment

The Quality Assessment of Diagnostic Accuracy Studies (QUADAS)-2 tool was adopted to evaluate the quality of references prior to the diagnostic meta-analysis (43). The evaluation system comprised two parts: bias evaluation and applicability. Specifically, the bias evaluation consisted of case selection, index tests, reference standards, and flow and timing, and the applicability evaluation of case selection, trials to be evaluated and gold standard. Each item could be classified as low risk, high risk and unknown, with the corresponding scores of 1, 0, and 0, respectively. The total score of ≥4 (with a full score of 7) indicated that the quality of literature research was high. The case-control study was evaluated according to 8 items of the Newcastle-Ottawa Scale (NOS scale) (44), categorized into study selection, comparability, and outcome. The total rated score of ≥5 (with a full score of 9) suggested that the quality of literature research was high.

### Statistical Analysis

Statistical analysis was carried out using MetaDiSc 1.4 and STATA 12.0 software, and the combined-effect indices involved sensitivity, specificity, positive likelihood ratio (PLR), negative likelihood ratio (NLR), diagnostic odds ratio (DOR), AUC, HR and 95% CIs. The difference in the merged area under the curve (AUC) between groups was compared by Z test. Spearman correlation coefficients were calculated to evaluate the threshold effect using MetaDiSc 1.4, while Cochran’ Q and *I* ^2^ tests to assess the non-threshold effect using STATA 12.0. The level of significance was set at *p* < 0.01 or *I* ^2^ > 50%. When there was no heterogeneity between studies, the statistics could be merged by the fixed-effect model; and when heterogeneity appeared, the statistics would be combined using the random-effect model. The sources of heterogeneity were deeply traced by a sensitivity analysis and a meta-regression test. Publication bias was judged by Deek’s quantitative funnel plot, as well as Begg’s and Egger’s tests, and the significant level was set at *p* < 0.1. The trim-and-fill method was adopted to assess the possible effect of publication bias (45).

## Results

### Study Selection and Data Characteristics

**FIGURE 1:**
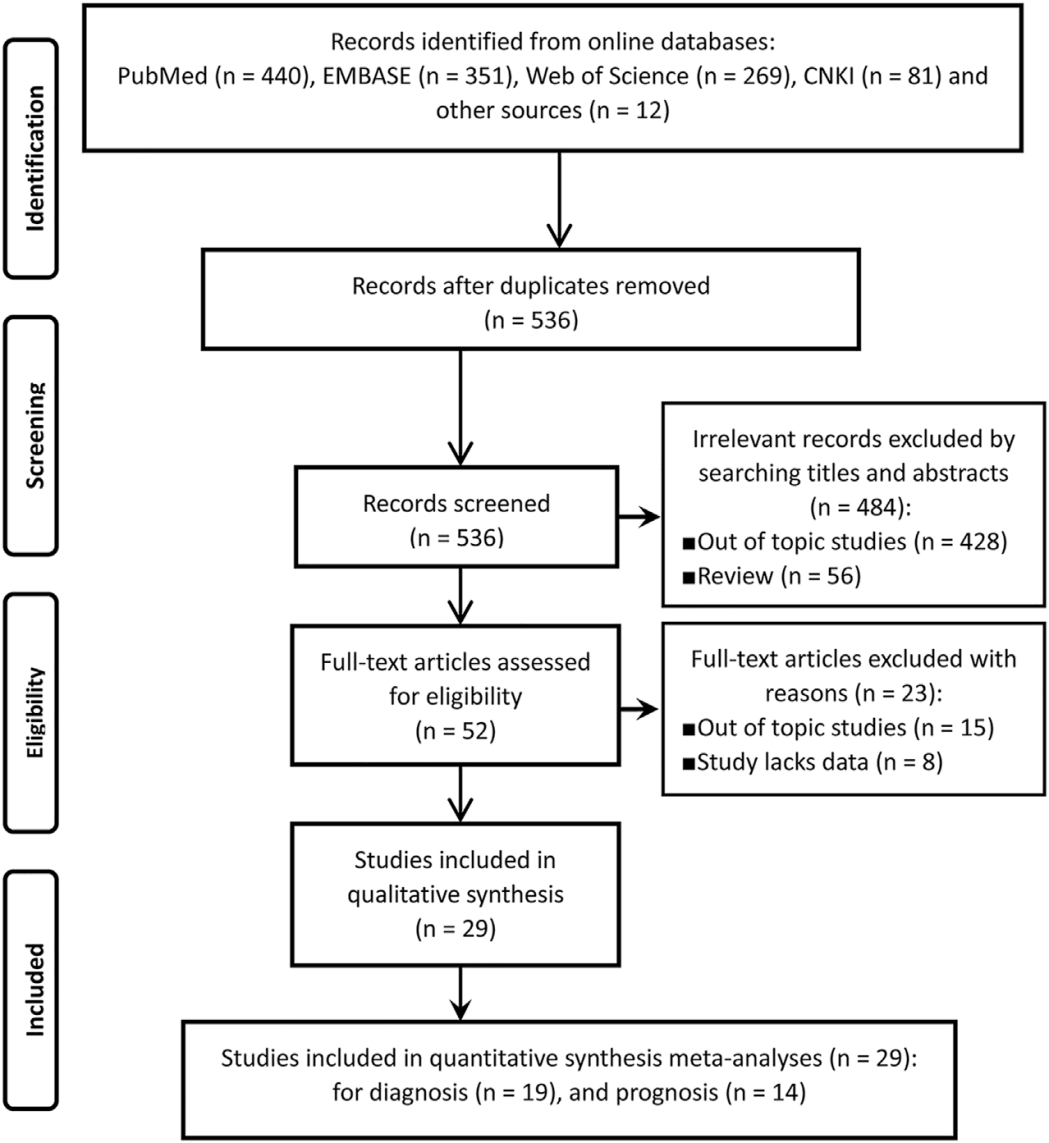
The flow chart of literature inclusion and exclusion process according to the PRISMA statement.

Of the initially screened 536 references from the aforesaid online databases according to the retrieval strategy, 29 articles (19 for diagnosis and 14 for prognosis) (14–42) that met the inclusion and exclusion criteria were eventually enrolled for our meta-analysis. The literature retrieval process was depicted in Figure 1.

The basic characteristics of the 29 articles (14–42) were summarized in Tables 1, 2. A total of 2639 CRC patients and 1471 matched controls were enrolled. The CRC cases were confirmed by pathological examinations and the included control group encompassed healthy subjects and paracancerous controls. All tissue and plasma samples were obtained preoperatively without any other treatment. The included individuals in our study included Asians and Caucasians, and 34 circRNAs were involved in the meta-analysis, of which 25 acting as oncogenes were up-regulated and 9 as tumor-suppressor genes were down-regulated in CRC. The expression levels of circRNAs in CRC were determined by RT-qPCR or RNA sequencing, and *GAPDH*, *18srRNA*, or *β-actin* mRNAs were used as internal reference genes. Of the 15 included studies on the prognosis of CRC, 9 provided available HRs and 95% CIs, 6 offered relevant data that could indirectly calculate the indices by formulas or prognosis curves. The median follow-up time varied from 1 to 39 months.

**TABLE 1:** Basic characteristics of the included studies for assessing diagnostic performances of circRNAs in CRC.

Study	Ethnicity	CRC number	Control number	Control type	Sample type	CircRNA name	Expression status	Measure method	Reference gene	Cut-off setting	AUC
Hsiao K 2017 (16)	Taiwan	131	76	PNC	Tissue	*circCCDC66*	Increased	RNA sequencing	*GAPDH*	/	0.88
Ji W 2018 (17)	Chinese	64	64	PNC	Tissue	*hsa_circ_0001649*	Increased	qRT-PCR	*GAPDH*	0.278	0.857
Li J 2018 (18)	Chinese	101	101	PNC	Tissue	*hsa_circ_0000711*	Decreased	qRT-PCR	*GAPDH*	ΔCt: 3.37	0.81
Li X 2019 (20)	Chinese	60	60	Healthy individuals	Plasma	*circVAPA*	Increased	qRT-PCR	*18S rRNA*	Median expression level of circVAPA	0.724
Ruan H 2019 (21)	Chinese	35	35	Adjacent normal tissues	Tissue	*hsa_circ_0002138*	Decreased	qRT-PCR	*β-actin*	0.005866	0.7249
Wang J 2018 (23)	Chinese	102	102	PNC	Tissue	*hsa_circ_0000567*	Decreased	qRT-PCR	*18S rRNA, GAPDH*	0.4714	0.8653
Wang F 2018 (22)	Chinese	46	46	PNC	Tissue	*hsa_circ_0014717*	Decreased	qRT-PCR	*GAPDH*	Unclear	0.6830
Wang X 2015 (24)	Chinese	62	62	PNC	Tissue	*hsa_circ_001988*	Decreased	qRT-PCR	*GAPDH*	6.04	0.7880
Zhang W 2018 (29)	Chinese	121	46	Healthy individuals	Plasma	*hsa_circ_0007534*	Increased	qRT-PCR	*GAPDH*	1.255	0.7800
Zhuo F 2017 (31)	Chinese	122	122	PNC	Tissue	*hsa_circRNA0003906*	Decreased	qRT-PCR	*GAPDH*	Unclear	0.8180
Zhang P 2017 (28)	Chinese	170	170	Normal colorectal tissue samples	Tissue	*hsa_circRNA_104700*	Decreased	qRT-PCR	*GAPDH*	10.753	0.6990
						*hsa_circRNA_103809*	Decreased	qRT-PCR	*GAPDH*	13.9	0.6160
Liu F 2021 (32)	Chinese	30	30	PNC	Tissue	*hsa_circ_0084927*	Increased	qRT-PCR	*β-actin*	Unclear	0.8060
Wang J 2021 (33)	Chinese	43	43	PNC	Tissue	*hsa_circ_0043278*	Decreased	qRT-PCR	*GAPDH*	Unclear	0.7100
Li J 2020 (34)	Chinese	102	80	Healthy individuals	Plasma	*hsa_circ_0001900*	Increased	qRT-PCR	*GAPDH*	1883 copies/ml	0.7220
Li J 2020 (34)	Chinese	102	80	Healthy individuals	Plasma	*hsa_circ_0001178*	Increased	qRT-PCR	*GAPDH*	582 copies/ml	0.7180
Li J 2020 (34)	Chinese	102	80	Healthy individuals	Plasma	*hsa_circ_0005927*	Increased	qRT-PCR	*GAPDH*	578 copies/ml	0.7840
Alkhizzi B 2021 (35)	Saudi Arabia	42	32	Healthy individuals	Plasma	*circMETTL3*	Increased	qRT-PCR	*GAPDH*	≥0.00296	0.6946
Alkhizzi B 2021 (35)	Saudi Arabia	42	32	Healthy individuals	Plasma	*circUSP3*	Increased	qRT-PCR	*GAPDH*	≥0.00493	0.6280
Mai S 2021 (36)	Chinese	148	148	Healthy volunteers	Serum	*circ_PVT1*	Increased	qRT-PCR	*GAPDH*	Unclear	0.8389
Mai S 2021(36)	Chinese	148	148	Healthy volunteers	Serum	*hsa_circ_001569*	Increased	qRT-PCR	*GAPDH*	Unclear	0.9016
Xu Y 2021 (37)	Chinese	60	60	PNC	Tissue	*hsa_circ_002178*	Increased	qRT-PCR	*GAPDH*	Unclear	0.8635
Wang J 2021 (38)	Chinese	84	84	PNC	Tissue	*circSPARC*	Increased	qRT-PCR	*18S rRNA*	Unclear	0.8613
Song Y 2021 (39)	Chinese	122	80	Healthy individuals	Plasma	*hsa_circ_0001821*	Increased	qRT-PCR	*GAPDH*	Unclear	0.815

AUC, area under the curve; GAPDH, reduced glyceraldehyde-phosphate dehydrogenase; qRT-PCR, quantitative reverse transcription-polymerase chain reaction; PNC, paired noncancerous counterparts.

**TABLE 2:** Basic characteristics of the included studies for the appraisal of prognostic effects of circRNAs on CRC.

Study	Ethnicity	Total CRC	CircRNA expression		Sample type	CircRNA profiling	Expression status	Survival indicator	Follow-up time
			High	Low					
Fang G 2018(14)	China	44	24	20	Tissue	*circRNA_100290*	Increased	OS	Unclear
Ge Z 2018(15)	China	63	/	/	Tissue	*CircMTO1*	Decreased	OS	Unclear
Hsiao KY 2017(16)	China	131	/	/	Tissue	*circCCDC66*	Increased	OS	Unclear
Li J 2018(18)	China	101	50	51	Tissue	*hsa_circ_0000711*	Decreased	OS	Medain: 39 months
Li R 2019(19)	China	58	/	/	Tissue	*hsa_circRNA_102958*	Increased	OS	Unclear
Yuan Y 2018(26)	China	32	15	17	Tissue	*circ_0026344*	Decreased	OS	Unclear
Wang F 2018 (22)	China	46	23	23	Tissue	*hsa_circ_0014717*	Decreased	OS	Intervals: 1–3 months
Weng W 2017(25)	China	153	76	77	Tissue	*ciRS-7*	Increased	OS	Unclear
Weng W 2017(25)	China	165	89	76	Tissue	*ciRS-7*	Increased	OS	Unclear
Zheng X 2019 (30)	China	100	/	/	Tissue	*circPPP1R12A*	Increased	OS	Unclear
Zeng K 2018(27)	China	178	89	89	Tissue	*circHIPK3*	Increased	OS	Unclear
Zhang W 2018(29)	China	112	72	40	Tissue	*hsa_circ_0007534*	Increased	OS	Unclear
Karousi P 2020 (40)	Greece	151	73	78	Tissue	*circ-BCL2L12-1*	Increased	OS	Medain: 52 months
Wang G 2021 (41)	China	46	24	22	Tissue	*circDUSP16*	Increased	OS	Unclear
Gao L 2021 (42)	China	169	90	79	Tissue	*circCOG2*	Increased	OS	Unclear
Song Y 2021 (39)	China	102	51	51	Plasma	*hsa_circ_0001821*	Increased	OS	Unclear

CRC, colorectal cancer; OS, overall survival.

### Heterogeneity Test

The Spearman correlation coefficient analysis showed a *p* value of 0.356 (Spearman correlation coefficient: −0.267) in diagnostic meta-analysis, suggesting that there was no heterogeneity resulted from the threshold effect. A *p* value of <0.001 and *I* ^2^ of 97.46% for the overall combined diagnostic effect were presented in Cochran’ Q and *I* ^2^ tests, indicating that substantial heterogeneity existed in the non-threshold effect. No heterogeneity was observed in the combined prognostic meta-analysis (oncogenic circRNAs: *I* ^2^ = 0.0%, *p* = 1.000; adjusted effect of the tumor-suppressor circRNAs: *I* ^2^ = 0.0%, *p* = 0.994).

### Bias Risk Assessments for the Included Studies

For the diagnostic meta-analysis, the QUADAS-2 scale was used to assess the risk of bias. As a result, the rated scores of all 19 articles were higher than 4 (Table 3), suggesting that the overall quality of the included studies was high. Consistently, the NOS scale showed high scores of over 6 in the observational studies (Table 4), indicating the high quality of the included case-control studies.

**TABLE 3:** The study quality and the bias risk in studies on the diagnostic efficacy of circRNAs were assessed by the QUADAS II checklist.

	Risk of bias				Concerns regarding applicability			Summed scores
Study	Patient selection	Index test	Reference standard	Flow and timing	Patient selection	Index test	Reference standard	
Hsiao KY 2017 (16)	Low	Unclear	Low	Unclear	Unclear	Low	Low	4
Ji WX 2018 (17)	Low	Unclear	Low	Unclear	Unclear	Low	Low	4
Li J 2018 (18)	Low	Unclear	Low	Unclear	Unclear	Low	Low	4
Li XN 2019 (20)	Low	Low	Low	Low	Unclear	Low	Low	6
Ruan H 2019 (21)	Low	Unclear	Low	Unclear	Unclear	Low	Low	4
Wang J 2018 (23)	Low	Unclear	Low	Low	Unclear	Low	Low	5
Wang F 2018 (22)	Low	Unclear	Low	Unclear	Unclear	Low	Low	4
Wang X 2015 (24)	Low	Unclear	Low	Low	Unclear	Low	Low	5
Zhang W 2018 (25)	Low	Low	Low	Low	Unclear	Low	Low	6
Zhuo F 2017 (31)	Low	Low	Low	Low	Unclear	Low	Low	6
Zhang P 2017 (28)	Low	Unclear	Low	Unclear	Unclear	Low	Low	4
Liu F 2021 (32)	Low	Unclear	Low	Unclear	Unclear	Low	Low	4
Wang J 2021(33)	Low	Unclear	Low	Unclear	Unclear	Low	Low	4
Li J 2020(34)	Low	Low	Low	Low	Unclear	Low	Low	6
Alkhizzi B 2021(35)	Low	Low	Low	Low	Unclear	Low	Low	6
Mai S 2021(36)	Low	Unclear	Low	Unclear	Unclear	Low	Low	4
Xu Y 2021(37)	Low	Unclear	Low	Unclear	Unclear	Low	Low	4
Wang J 2021(38)	Low	Unclear	Low	Unclear	Unclear	Low	Low	4
Song Y 2021(39)	Low	Unclear	Low	Unclear	Unclear	Low	Low	4

QUADAS, Quality Assessment for Studies of Diagnostic Accuracy.

**TABLE 4:** The study quality and the bias risk in case-control studies were rated by the NOS scale.

Study	Summed scores	Cohort selection				Comparability	Outcome ascertainment		
		Representativeness of the exposed cohort	Selection of the non-exposed cohort	Ascertainment of exposure	Demonstration that outcome of interest was not present at start of study	Comparability of cases and controls on the basis of the design or analysis	Assessment of outcome	Was follow-up long enough for outcomes to occur	Adequacy of follow up of cohorts
Fang G 2018 (14)	6	1	1	1	1	1	1	0	0
Ge Z 2018 (15)	6	1	1	1	1	1	1	0	0
Hsiao KY 2017 (16)	6	1	1	1	1	1	1	0	0
Li J 2018 (18)	8	1	1	1	1	1	1	1	1
Li R 2019 (19)	6	1	1	1	1	1	1	0	0
Yuan Y 2018 (26)	6	1	1	1	1	1	1	0	0
Wang F 2018 (22)	8	1	1	1	1	1	1	1	1
Zeng K 2018 (27)	6	1	1	1	1	1	1	0	0
Weng W 2017 (25)	6	1	1	1	1	1	1	0	0
Zheng X 2019 (30)	6	1	1	1	1	1	1	0	0
Zeng K 2018 (27)	6	1	1	1	1	1	1	0	0
Zhang W 2018 (29)	6	1	1	1	1	1	1	0	0
Karousi P 2020 (40)	8	1	1	1	1	1	1	1	1
Wang G 2021 (41)	6	1	1	1	1	1	1	0	0
Gao L 2021(42)	6	1	1	1	1	1	1	0	0
Song Y 2021(39)	6	1	1	1	1	1	1	0	0

### Diagnostic Efficiency

**FIGURE 2:**
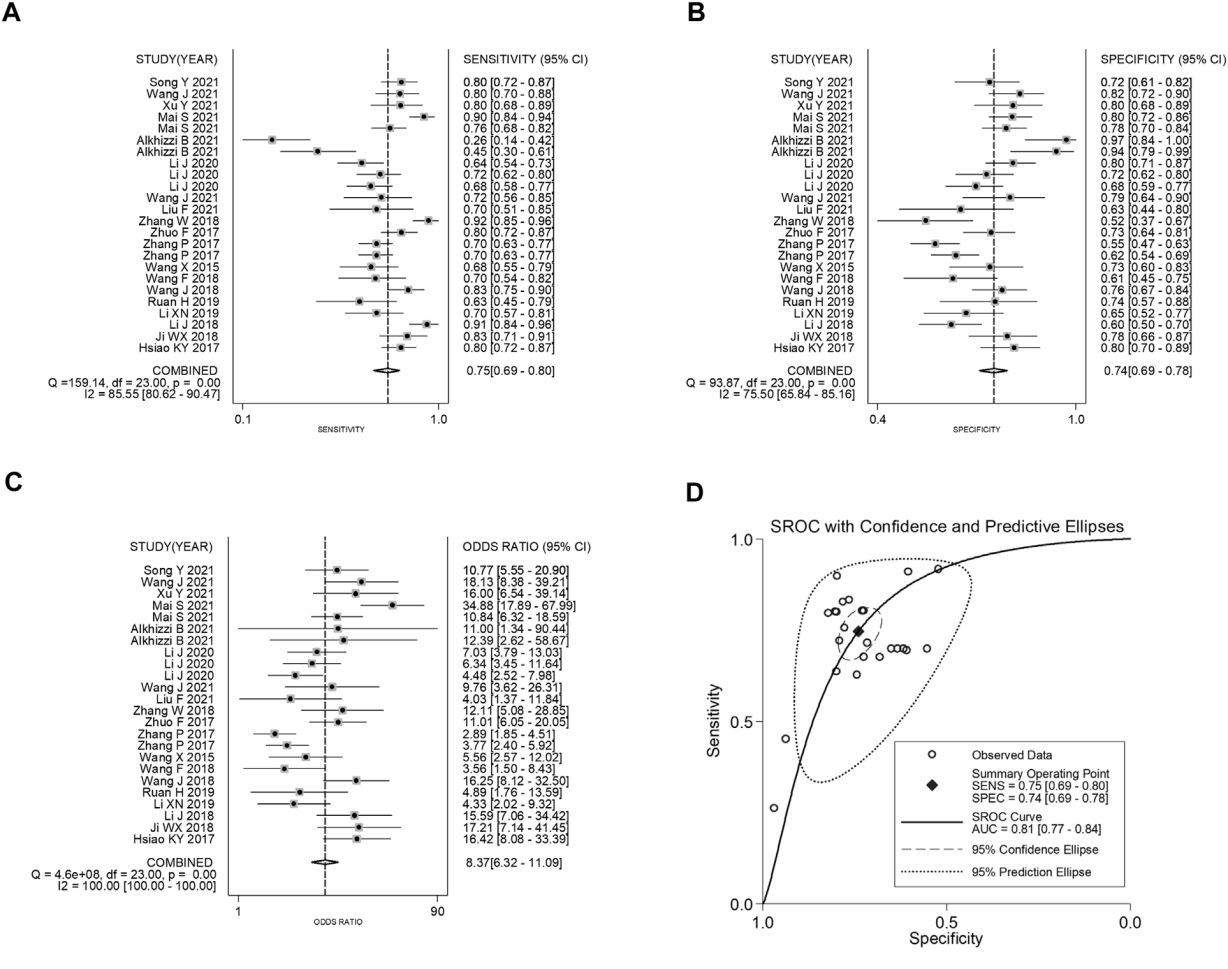
Forest plots of pooled **(A)** sensitivity, **(B)** specificity, **(C)** DOR, and **(D)** AUC for the diagnostic effect of circRNAs in CRC.

The forest plots showed that the pooled sensitivity, specificity, PLR, NLR, DOR and AUC of circRNAs in the diagnosis of CRC were 0.75 (95% CI: 0.69–0.80), 0.74 (95% CI: 0.69–0.78), 2.87 (95% CI: 2.46–3.34), 0.34 (95% CI: 0.28–0.42), 8.37 (95% CI: 6.32–11.09) and 0.81, as shown in Figure 2. Subgroup analysis showed that the AUC of up-regulated circRNAs in the diagnosis of CRC was higher than that of down-regulated circRNAs (AUC: 0.83 versus 0.77; Z test, *p* < 0.05), and the former had higher diagnostic specificity and DOR (Table 5). Analysis based on different control sources showed that the efficacy of circRNA expression profile was higher in distinguishing CRC from healthy differentiation than its ability to distinguish CRC from PNC (Table 5). Analysis based on different sample type and reference gene showed that the efficacy of circRNA abnormal expression profile based on issue was equivalent to that of ACU based on plasma/serum; GAPDH based testing and non-GAPDH based testing could also obtain similar results (Table 5).

**TABLE 5:** Subgroup study of the diagnostic efficacy of circRNAs in CRC.

Analysis variable	Sensitivity (95% CI)	Specificity (95% CI)	PLR (95% CI)	NLR (95% CI)	DOR (95% CI)	AUC	Heterogeneity (*I* ^2^)
S (%)ample Type							
Tissue	0.77 (0.74–0.79)	0.69 (0.67–0.72)	2.66 (2.17–3.27)	0.33 (0.27–0.41)	8.37 (5.55–12.60)	0.81	77.4
Plasma/serum	0.74 (0.71–0.77)	0.75 (0.72–0.78)	2.79 (2.25–3.47)	0.37 (0.26–0.52)	9.14 (6.00–13.92)	0.82	68.1
Control sources							
CRC *vs.* PNC	0.76 (0.74–0.79)	0.69 (0.66–0.72)	2.60 (2.15–3.16)	0.34 (0.28–0.42)	8.00 (5.43–11.79)	0.80	76.3
CRC *vs.* Healthy individuals	0.75 (0.72–0.78)	0.75 (0.72–0.78)	2.91 (2.39–3.54)	0.35 (0.24–0.50)	10.05 (6.86–14.74)	0.83	63.6
circRNA expression status							
Up-regulated circRNAs	0.76 (0.73–0.78)	0.76 (0.73–0.78)	3.01 (2.51–3.61)	0.33 (0.25–0.44)	10.34 (7.40–14.45)	0.83	63.8
Down-regulated circRNAs	0.75 (0.72–0.78)	0.66 (0.63–0.69)	2.31 (1.88–2.84)	0.37 (0.28–0.48)	6.60 (4.12–10.55)	0.77	76.6
Reference gene							
*GAPDH* based testing	0.75 (0.73–0.77)	0.71 (0.69–0.73)	2.71 (2.29–3.21)	0.34 (0.27–0.43)	8.85 (6.35–12.33)	0.81	75.8
Non-*GAPDH* based testing	0.76 (0.71–0.81)	0.74 (0.69–0.79)	2.75 (1.96–3.86)	0.35 (0.24–0.51)	8.00 (4.05–15.81)	0.80	69.1

AUC, area under the curve; CRC, colorectal cancer; PNC, Paired noncancerous counterparts; PLR, positive likelihood ratio; NLR, negative likelihood ratio; DOR, diagnostic odds ratio.

### Prognostic Analysis

**FIGURE 3:**
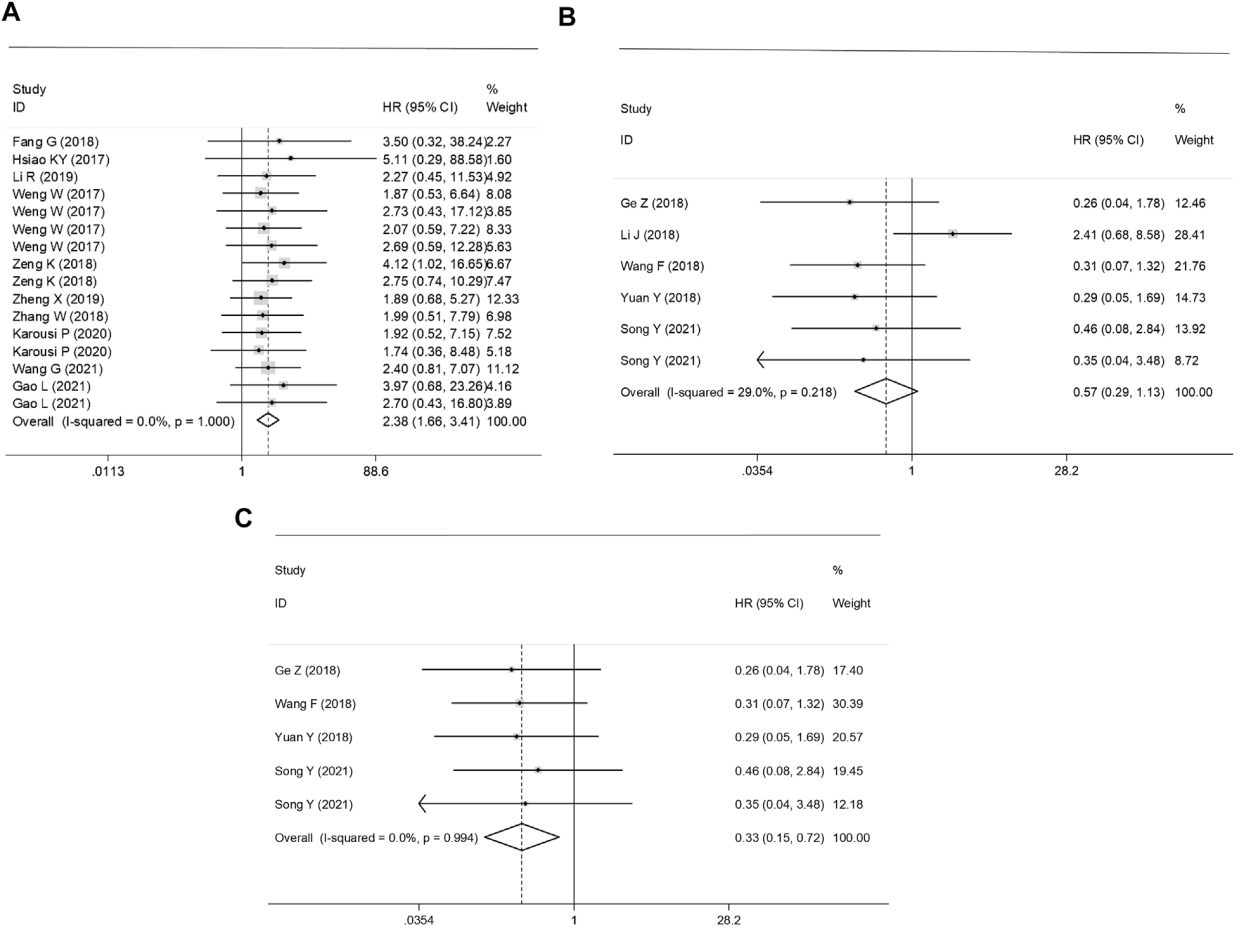
Forest plots of the prognostic effect of **(A)** up-regulated, **(B)** un-adjusted down-regulated circRNAs and **(C)** adjusted down-regulated circRNAs on survival in CRC patients.

The subgroup classification was conducted according to the gene functions of circRNAs. The prognosis analysis showed that high expression levels ofup-regulated circRNAs were associated with the poor OS in GC patients (HR = 2.38, 95% CI: 1.66–3.41, *p* = 0.000), whereas the total survival time in GC patients with high expressed down-regulated circRNAs was significantly prolonged (an outlier eliminated adjusted HR = 0.33, 95% CI: 0.15–0.72, *p* = 0.006) (Figure 3).

### Sensitivity Analysis and the Meta-Regression Test

**FIGURE 4:**
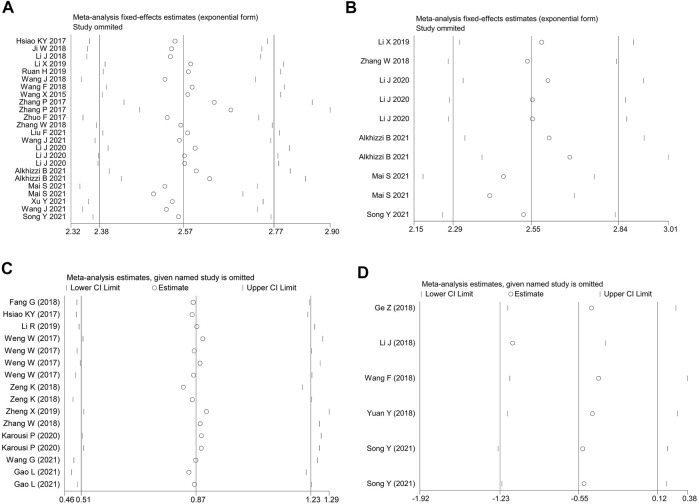
The influence analysis of the homogeneity among the included studies. **(A)** the overall diagnostic efficacy of the studies. **(B)** the diagnostic efficacy of plasma/serum-based circRNA testing. **(C)** up-regulated and **(D)** down-regulated circRNAs in predicting the OS in CRC patients.

Sources of heterogeneity were traced by a sensitivity analysis, and no outliers were identified in the diagnostic meta-analyses and the prognostic effect for the plasma/serum-based circRNA testing (Figures 4A–C). However, it was found that data of one independent study of the prognostic effect was identified as an outlier (18) (Figure 4D). After the removal of the outlier, the *p* value of Cochran’ Q test varied from 0.000 to 0.961, and *I* ^2^ decreased to 0.0% from 29.0%. All this suggested that the inclusion of outliers for statistical consolidation could be considered as one of the important reasons for the heterogeneity of the analysis results. Thus, the adjusted HR of the down-regulated circRNAs was calculated after an elimination of the outlier (Figure 3C).

On the other hand, a meta-regression test was performed to analyze factors comprising the quality of the study, the type of study, the type of specimen, the number of cases and the number of control groups. The results showed that these factors were not the source of heterogeneity among the studies with nonsignificant differences (data not shown).

### Publication Bias

**FIGURE 5:**
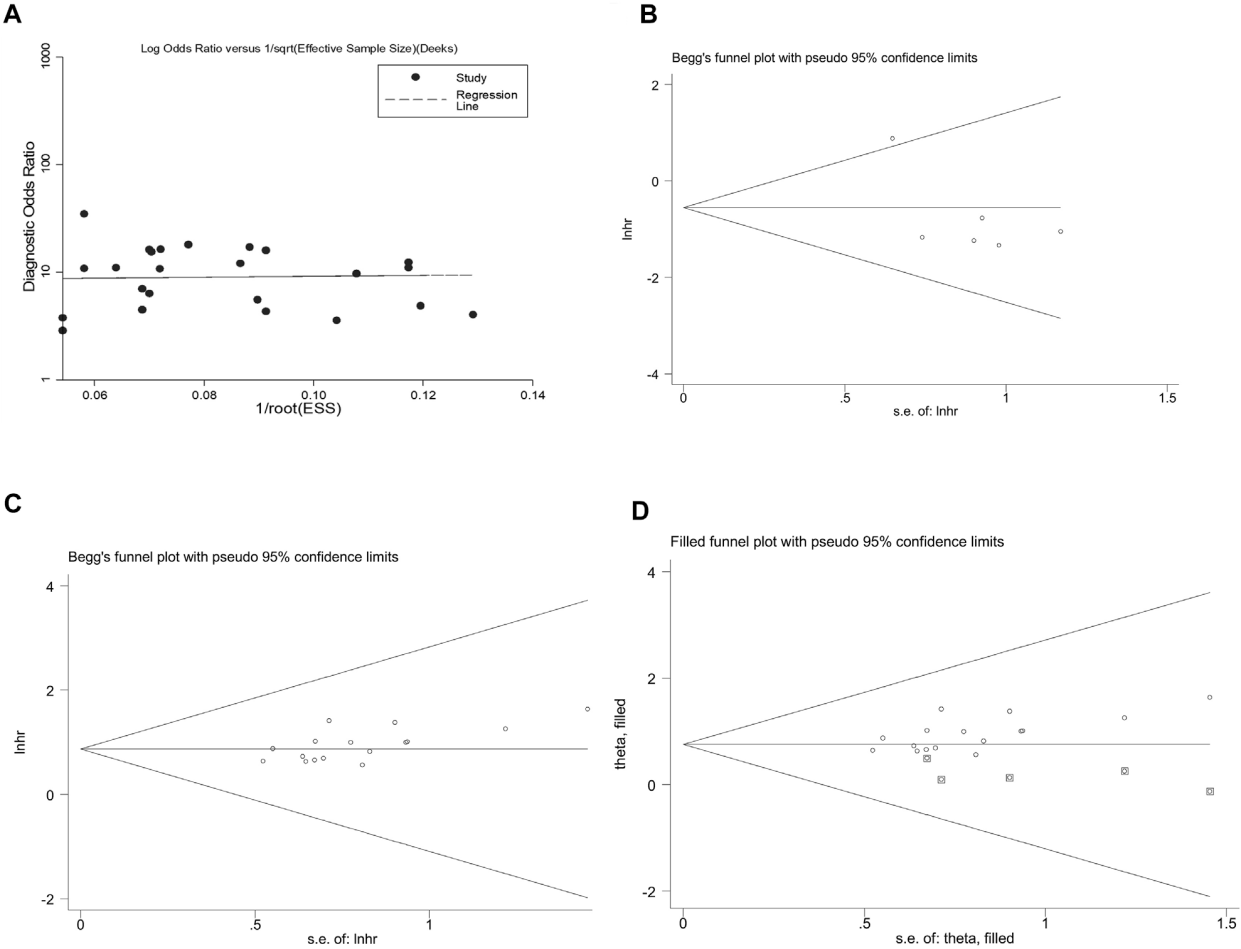
Appraising the publication bias among the included studies. **(A)** the overall diagnostic effect assessed by the Deek’s funnel plot. Begg’s test for the effects of **(B)** down-regulated and **(C)** up-regulated circRNAs in predicting the OS in CRC patients. **(D)** funnel plot of the trim-and-fill method shows no significant impacts on the combined effect of up-regulated circRNAs in CRC.

Assessment of the publication bias is shown in Figure 5. We observed publication bias in the combined prognostic effect forup-regulated circRNAs (Egger’s test, *p* = 0.021) (Figure 5C). The missing studies were adjusted through the trim-and-fill method (Figure 5D). Nevertheless, the pooled analysis incorporating the hypothetical studies (moment-based estimate of between studies variance = 0.000, *p* = 1.000) altered slightly from the unadjusted ones (moment-based estimate of between studies variance = 0.000, *p* = 1.000), suggesting that the bias did not have a significant impact on the combined effect ofup-regulated circRNAs in CRC.

## Discussion

CRC is a common malignant tumor of the digestive tract, with nearly 0.6 million newly diagnosed cases across the world every year (1,2). Early detection, diagnosis and treatment of the disease can maximize the survival of the patients (3,5,7). CircRNAs as a class of coding/non-coding competitive endogenous RNA, are characterized by a closed covalent continuous ring structure without the 5′-3′ structure and a poly (A) tail (8,9). A large number of circRNAs existing in mammals affect their functional expressions by binding to miRNAs and other target molecules at the transcriptional or post-transcriptional level (10,11). Although mechanisms behind their biofunctions have not been completed expounded, increasing evidences have shown that circRNAs are associated with multiple diseases, especially malignant tumors (12,13). In recent years, it has been found that expression profiles of circRNAs in CRC cancerous tissues are significantly different from those in the corresponding paracancerous tissues, suggesting that the abnormally expressed circRNAs can be critical in the onset and development of this malady. And they are expected to become new biomarkers in the early diagnosis and treatment of CRC (14–31). Herein, we conducted a pair-wire meta-analysis, and assessed the diagnostic and prognostic significance of abnormally expressed circRNAs in CRC.

In our study, expression levels of circRNAs present clinical values in its diagnosis, with the sensitivity and specificity of 0.75 and 0.74 respectively and the corresponding AUC of 0.81. DOR, the rate of true positive to false positive, is another important indice to evaluate the effectiveness of circRNA-based diagnostic tests (46). A DOR of less than 1 indicates the very low efficiency in the diagnosis of CRC (46). However, a prominent outcome of 8.37 was presented in our study, suggesting the high efficiency of circRNA-based diagnosis. In addition, the pooled PLR of 2.86 means the probability of positive circRNA expressions in CRC patients is approximately 5 times higher than that in controls. The pooled NLR of 0.34 indicates that only 34% of the negative results of circRNA tests are false negative. These findings fully prove that circRNA detection can be applied in the early diagnosis of CRC. As proven by two previous studies, the sensitivity, specificity and AUC of combined circRNA expression profiles in the diagnosis of all malignant tumors are 0.72, 0.74, and 0.79 (47), and 0.79, 0.73, and 0.83 in the diagnosis of gastrointestinal cancers (48). These findings are consistent with our results.

The expression characteristics and sample types of circRNAs have been analyzed by subgroup analyses. We find that up-regulated circRNAs are more effective in diagnosing CRC than down-regulated circRNAs, acting as oncogenes. The analysis based on the sample type indicates that the high kurtosis in the newly diagnosed CRC patients is more beneficial to the detection. The AUC in tissue-derived circRNAs is higher than that in plasma-derived ones, suggesting that the tissue-based circRNA tests may be more accurate than the plasma-related tests; however, this also may attribute to a higher expression peak of circRNAs in cancerous tissues. In spite of this, the number of samples in the subgroup analysis is lower than that of the whole, and the conclusion needs to be confirmed by large sample size trials with later-stage patients.

As circRNAs have been proven to be associated with the prognosis of malignant tumors, systematical evaluations for the prognostic efficacy of circRNA expressions in lung cancer and hepatocellular carcinoma have been conducted (49,50). The results show that the higher the levels of up-regulated circRNAs are, the worse the prognosis of the cancer patients will be. Evidences show that expressions of down-regulated circRNAs in hepatocellular carcinoma are associated with a dismal survival time. These patients with high expressed circRNAs are more likely to extend the OS time. It can be seen that biological functions of different expression states of circRNAs in malignant tumors are distinct. Therefore, we evaluated circRNA as a potential prognostic biomarker of CRC. According to the principle of different biofunctions of circRNAs corresponding to their types, we have divided the circRNA expression profile into two groups: oncogenes and tumor-suppressor ones. The survival analysis showed that the total survival time of the patients with high-expressed up-regulated circRNAs was significantly shorter than that of patients with low-expression levels. And the CRC patients with low expressions of up-regulated circRNAs and high expressions of tumor-suppressor circRNAs significantly presented favorable prognosis, suggesting that circRNA profiling can be used as indicators for evaluating and monitoring the prognosis of CRC.

The main sources of heterogeneity in a meta-analysis consist of the threshold effect and the non-threshold effect. Results of the Spearman correlation coefficient analysis indicate that the heterogeneity in the overall merger statistics and subgroup analyses mainly result from the threshold effect, which may attribute to different boundary values or cut-off values. In this study, the relative quantitative cut-off values and internal reference genes for circRNAs are different, which can be one of the main causes of heterogeneity. Besides, our study has discussed the possible factors leading to heterogeneity by the sensitivity analysis and the meta-regression analysis. We identified an outlier study in the sensitivity analysis and its impact on the prognostic effect was verified. However, the meta-regression test has ruled out factors including the quality of the study, the type of study, the type of specimen, the number of cases and the number of control groups as the possible sources of heterogeneity among the studies.

As the biofunctions of circRNAs in CRC have been clarified above, some limitations still exist in our study. Firstly, the types of circRNA molecules and samples enrolled are not uniform, featuring a large heterogeneity among the studies. Secondly, the included subjects are predominantly Chinese population, which means certain population bias exists in the merged data. Thirdly, there are merely a handful of studies using plasma- or serum-derived circRNAs, and the relevant findings need to be verified by further researches.

## Conclusion

To sum up, circRNAs can be used as promising auxiliary biomarkers for the diagnosis and prognosis prediction of CRC. Our findings still need to be confirmed based on more high-quality trials.

## Data Availability

The datasets presented in this study can be found in online repositories. The names of the repository/repositories and accession number(s) can be found in the article/supplementary material.
